# Fluorescent Nanosensor Based on Molecularly Imprinted Polymers Coated on Graphene Quantum Dots for Fast Detection of Antibiotics

**DOI:** 10.3390/bios8030082

**Published:** 2018-09-05

**Authors:** Tongchang Zhou, Arnab Halder, Yi Sun

**Affiliations:** Department of Micro- and Nanotechnology, Technical University of Denmark, Ørsteds Plads, DK-2800 Kgs Lyngby, Denmark; tongz@nanotech.dtu.dk (T.Z.); arhal@nanotech.dtu.dk (A.H.)

**Keywords:** graphene quantum dots, molecularly imprinted polymers, tetracycline, fluorescence quenching

## Abstract

In this work, we developed a novel fluorescent sensor by combining molecularly imprinted polymers (MIPs) with graphene quantum dots (GQDs) for the determination of tetracycline (TC) in aqueous samples. Firstly, we developed a one-pot green method to synthesize GQDs as the fluorescent probes. GQDs with carboxyl groups or amino groups were fabricated. It was found that carboxyl groups played an important role in the fluorescence quenching. Based on these findings, the GQDs-MIPs microspheres were prepared using a sol-gel process. GQDs-MIPs showed strong fluorescent emission at 410 nm when excited at 360 nm, and the fluorescence was quenched in the presence of TC. Under optimum conditions, the fluorescence intensity of GQDs-MIPs decreased in response to the increase of TC concentration. The linear rage was from 1.0 to 10^4^ µg·L^−1^, and the limit of detection was determined to be 1 µg·L^−1^. The GQDs-MIPs also demonstrated high selectivity towards TC. The fluorescent sensor was successfully applied for the detection of TC in real spiked milk samples.

## 1. Introduction

Tetracycline (TC) is a kind of antibiotic commonly used in food-producing animals due to its broad-spectrum activity as well as its low cost. It also works as a growth promoter in animals. Recently, TC residues in food such as milk, eggs and meats have attracted a lot of attention, since its residues in food products can accumulate in human bodies through the food chain, and cause serious side effects such as dizziness, muscle pain or headache [1]. The overuse of these antibiotics is also believed to be responsible for the formation of antibiotic-resistant bacteria strains [2,3]. As such, the European Union has set the maximum residue limit (MRL) of TC in milk to be 100 µg·L^−1^, while the U.S. Food and Drug Administration (FDA)’s regulation is 300 ppb (around 300 µg·L^−1^) [4]. Conventionally, analytical techniques such as UV, HPLC or other techniques have been used to detect TC [5,6,7]. However, high cost and complicated operation procedures have limited their applications. Thus, a fast and facile method is needed to monitor the residue of TC in food samples.

Molecular imprinting is a technique for the formation of molecularly imprinted polymers (MIPs) with tailor-made binding sites complementary to the template molecules in shape, size and functional groups [8,9,10,11,12,13]. In particular, fluorescent MIP-based sensors offer a convenient solution for analyte detection due to the high sensitivity and selectivity. They have many advantages, such as short analysis time, ease of use, and small sample volume. One simple way to prepare fluorescent MIPs is adding fluorescent monomers to MIP during synthesis [14,15]. However, fluorescence monomer normally involves complicated synthesis work. Quantum dots (QDs) are semiconductor nanocrystals that can provide narrow and tunable emission spectra [16]. Compared with organic dyes, QDs have attracted much more attention due to photochemical stability and good water dispersibility. These properties make them appropriate as fluorescent probes. MIPs’ coated QDs sensors have been used for detection of amoxicillin [17], melamine [18], hemoglobin [19] and cytochrome c [20]. However, most work has used CdTe QDs, which displayed high cytotoxicity [21,22]. Therefore, environmentally friendly QDs are a better alternative and potentially safer to use in point-of-source applications.

Graphene quantum dots (GQDs) are a new class of carbon nanomaterial, which have lower toxicity and better biocompatibility than the traditional QDs [23,24]. GQDs have been directly used to detect cytochrome c [25]. By incorporating GQDs into the MIP matrix, the nanocomposites will possess both the high sensitivity of fluorescent probes and the selective recognition properties of MIPs. GQDs have been widely prepared with graphene oxide (GO) as a precursor via various methods. However, many methods for GQDs fabrication require strong acids, and long purification time. In addition, different fabrication and doping methods may result in different functional groups on the surface of GQDs, whereas the effects of functional groups on fluorescence quenching have not yet been studied [26,27]. Furthermore, although MIPs-coated GQDs have been reported as a nanosensor for detection of various compounds, detection of TC in food samples using GQDs-MIPs has seldom been demonstrated. 

In our work, an efficient green synthetic strategy was applied to prepare GQDs with different functional groups in a single step. We found that surface functional groups on GQDs played an important role in the fluorescence quenching. GQDs with carboxyl groups could be quenched by TC, while no quenching was observed for GQDs with amino groups. MIPs coated on GQDs with carboxyl groups were synthesized using eco-friendly reaction solvent (mixture of water and ethanol) and were used for the determination of template TC with high sensitivity. The relative fluorescence intensity of GQDs-MIPs decreased linearly with increasing TC in the concentration range of 1–10^4^ µg·L^−1^ with a detection limit of 1 µg·L^−1^. The GQDs-MIPs were also successfully applied to detect spiked TC in milk samples and gave recoveries from 85.3% to 103.3% with relative standard deviations of 3.7%–7.2%. These results demonstrated that GQDs-MIPs could potentially be used as a simple and fast responding fluorescence probe for sensitive and selective determination of TC.

## 2. Materials and Methods

### 2.1. Materials

All reagents were of analytical or HPLC grade and were used as received. Aminopropyltriethoxysilane (APTES), Tetraethoxysilane (TEOS), Ammonium hydroxide solution (28%–30%), hydrogen peroxide (H_2_O_2_, 30%), Gentamicin (GM) and amoxicillin (AM) and tetracycline (TC) were obtained from Sigma Aldrich. Nunclon 96-well flat-bottom black microwell plates were purchased from Thermo scientific, DK. 0.1% milk (Arla) was bought from a local supplier. The water used in the experiment was obtained from a Millipore (MilliQ) purification system.

### 2.2. Synthesis of Two Kinds of Graphene Quantum Dots (GQDs)

#### 2.2.1. Synthesis of Carboxylic Acid Functionalized Graphene Quantum Dots (GQDs-COOH)

To synthesize GQDs with the carbonyl group, 40 mg of graphene oxide precursor was mixed with 30 mL of H_2_O_2_ (5%). Then the mixture was transferred into a 50 mL Teflon-made autoclave chamber. The autoclave was put in an oven at 180 °C for 2 h. After cooling down to room temperature, the resulting solution was filtered using 0.2 µm filter paper to remove unreacted graphene oxide, and the resulting filtrate was collected and stored at room temperature. 

#### 2.2.2. Synthesis of Amino Functionalized Graphene Quantum Dots (GQDs-NH_2_)

Forty milligrams of graphene oxide precursor was mixed with 5 mL of H_2_O_2_ (30%), 5 mL of NH_4_OH (28%–30%) and 20 mL of water. The mixture was transferred into a 50 mL Teflon-made autoclave chamber. The reaction mixture in the autoclave was kept in an oven at 180 °C for 2 h. After cooling down to room temperature, the resulting solution was filtered using a 0.2 µm filter paper, and the resulting filtrate was collected. 

### 2.3. Preparation of GQDs-MIPs from Sol-Gel Process

Briefly, 15 mL of GQDs (3 mg) solution and 10 mL of ethanol were added into a flask. Then 80 µL of APTES were added and stirred for 2 h under vigorous stirring to allow the APTES to self-assemble onto the GQDs. Template TC (10 mg) was then dissolved in ultrapure water (10 mL) and added to the above solution. After stirring for 15 min, 100 µL of ammonia hydroxide solution (25%) was added, then 100 µL of TEOS and 10 mL of ethanol were added drop by drop. The reaction mixture was stirred at room temperature for 24 h.

The final products GQDs-MIPs were collected by centrifugation, then washed thoroughly with ethanol and MilliQ water. After each washing step, the supernatant was measured by UV to check whether there was TC residue in the solution. Typically the GQDs-MIPs were washed five times in order to completely remove the TC template. Non-imprinted particles (GQDs-NIPs) as a control were prepared similarly, except that the template was not added.

### 2.4. Characterization of GQDs-MIPs

TEM images were taken using a Tecnai T20 G2 (FEI, Hillsboro, OR, USA) transmission electron microscope. IR spectra were taken using a Spectrum 100 (PerkinElmer, Waltham, MA, USA). X-ray photoelectron spectroscopy (XPS) analysis was carried out by Thermo Scientific^TM^ K-Alpha+^TM^ XPS System with an Al K-Alpha (1486 eV) X-ray source.

### 2.5. Fluorescence Measurement of GQDs-MIPs 

TC with different concentrations (2, 20, 200, 2000 and 2 × 10^4^ µg·L^−1^, 150 µL) and GQDs-MIPs solution (150 µL, 1 mg·mL^−1^) were sequentially injected into each well of a Nunclon 96-well flat-bottom black microplate. Then, the fluorescence spectra were measured with an excitation wavelength of 360 nm, using a Spark^®^ multimode microplate reader (Tecan, Sweden). The fluorescent intensity at 410 nm was used for analysis.

For detection of TC in real samples, TC with the concentrations of 2, 20, 200, 2000 and 2 × 10^4^ µg·L^−1^ were spiked into the diluted milk (Arla 0.1%, diluted 200 folds). For each concentration, three samples were tested to get the standard deviation. The measurement was taken in the same manner as described above.

## 3. Results and Discussion

### 3.1. Green Synthesis of GQDs

**Figure 1:**
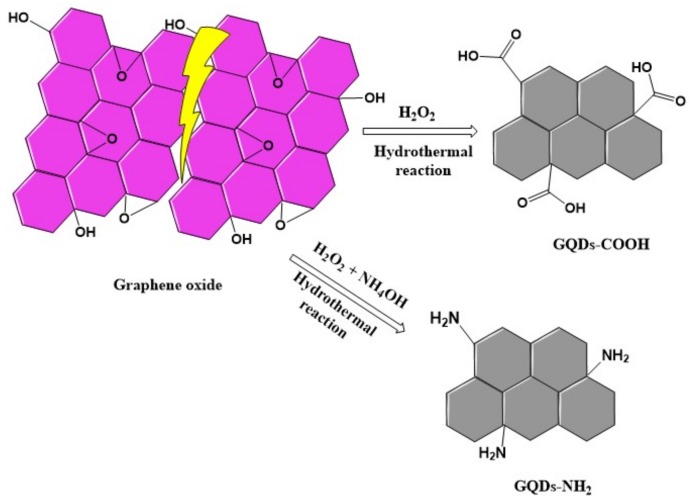
Illustration of the preparation of GQDs from graphene oxide.

It is of great importance to explore a mild, clean, and highly efficient approach for the synthesis of GQDs. Generally, GQDs were synthesized by traditional methods such as electrochemical exfoliation [28], chemical oxidation [29] and microwave-assisted methods [30]. Strong acids and chemicals are often involved as oxidants in most approaches. Some amount of chemicals are left as residues in the resulted GQDs solution, which need repeated washing steps to get pure GQDs. Herein, we introduced a new green and rapid preparation approach for GQDs using a low amount of hydrogen peroxide as an oxidant, as shown in Figure 1. As a degrading agent for graphene, H_2_O_2_ could cut down graphene into smaller sized graphene-based QDs. The byproducts are only H_2_O and CO_2_, and a post purification step is not needed.

### 3.2. Comparison of GQDs with Different Functional Groups

**Figure 2:**
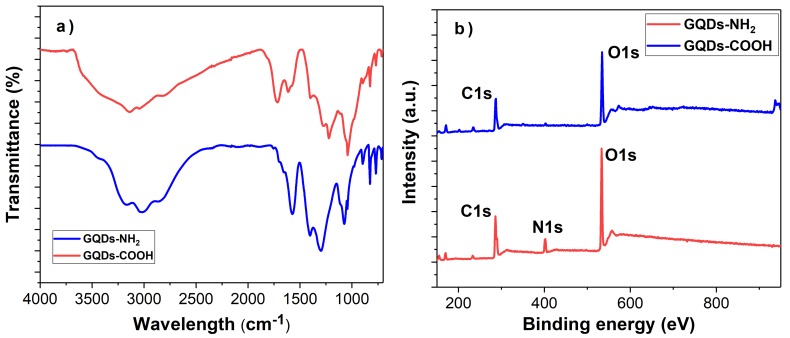

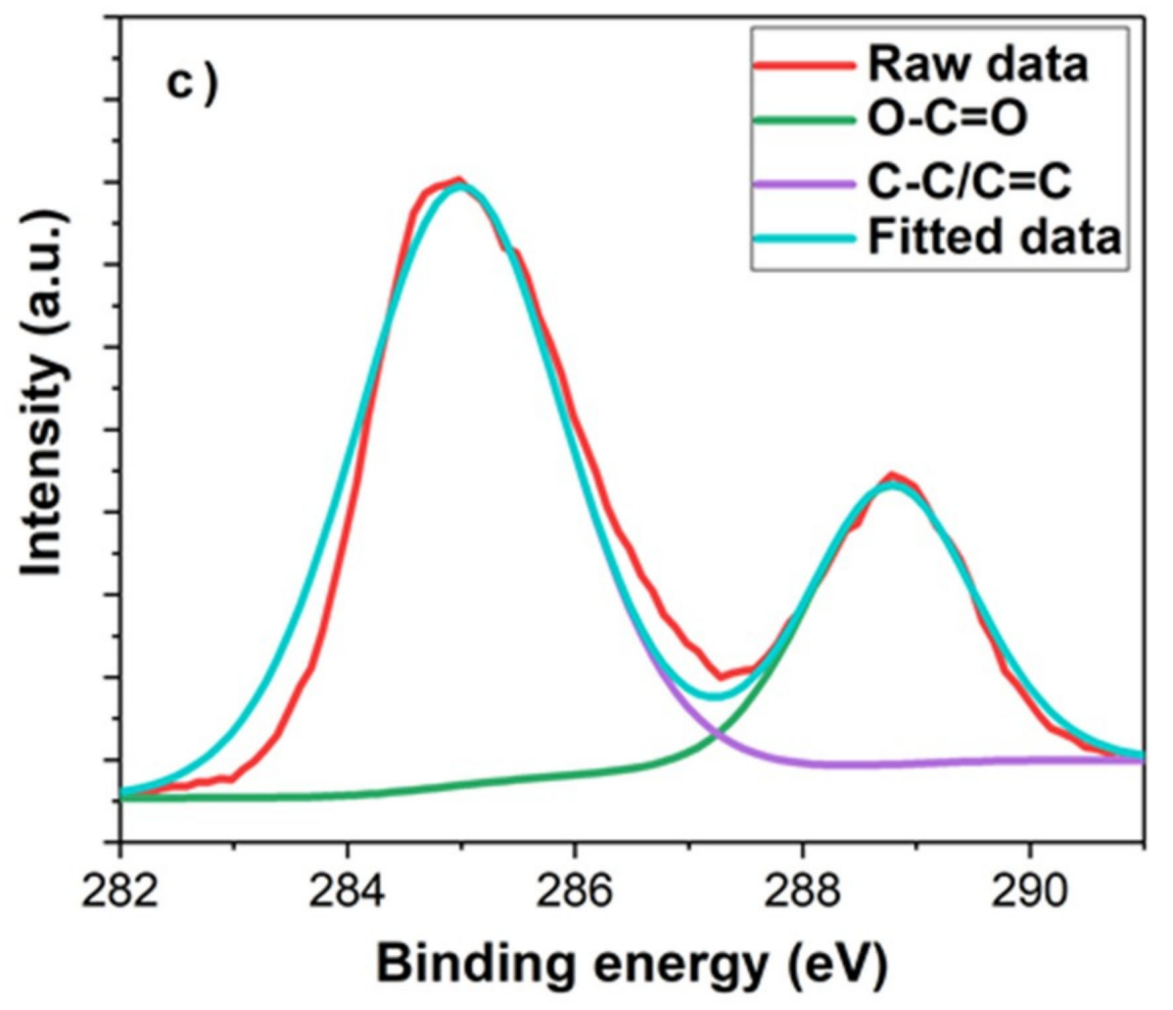
(**a**) FTIR of two types of GQDs. (**b**) XPS survey spectra for GQDs-COOH and GQDs-NH2. (**c**) The high resolution deconvoluted C 1s spectra for GQDs-COOH.

As carbon-based materials, GQDs have excellent properties, such as high surface area and low toxicity. In addition, surface functional groups can effectively tune their properties, which can extend their application areas. In this work, we successfully prepared GQDs with two kinds of functional groups. Figure 2a shows FT-IR spectra of the functional groups on the GQDs. The sharp peak appearing at 1720 cm^−1^ and 1226 cm^−1^ belonged to the C=O stretching and C-O stretching of carboxylic acid, respectively. In the meantime, a band appeared at 1576 cm^−1^ and was assigned to C-N stretching while the band appearing at 1291 cm^−1^ was assigned to the mixed vibration of C-N stretching and N-H bending. The detailed surface chemical bonding nature of GQDs-COOH and GQDs-NH_2_ were evaluated using XPS. As shown in Figure 2b, both of GQDs-COOH and GQDs-NH_2_ showed C 1s and O 1s peaks at around 286 eV and 532 eV. However, only a new peak was observed for GQDs-NH_2_ at around 400 eV, which was due to the presence of nitrogen. The C 1s spectra of GQDs-COOH was further deconvoluted into two peaks with binding energies at 284.9 and 288.7 eV, which correspond to sp2 and sp3 hybridized carbon (C-C/C=C) and O-C=O components respectively (Figure 2c).

**Figure 3:**
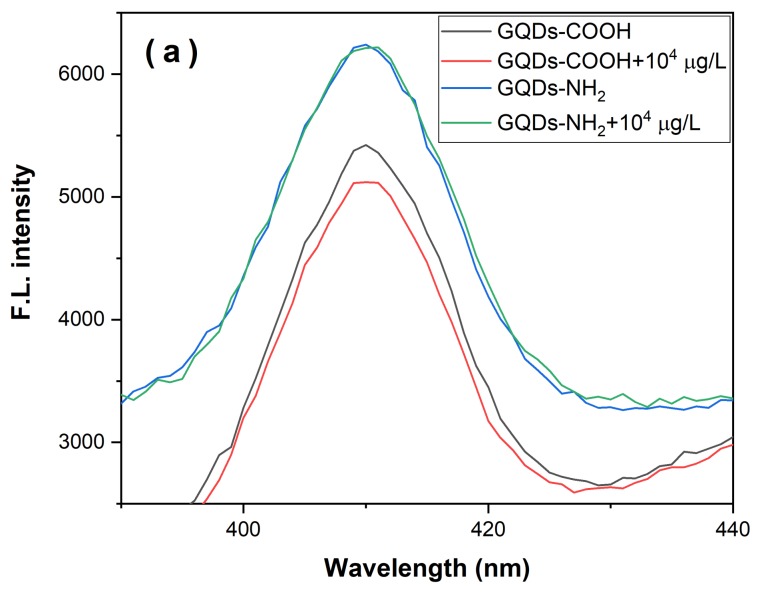

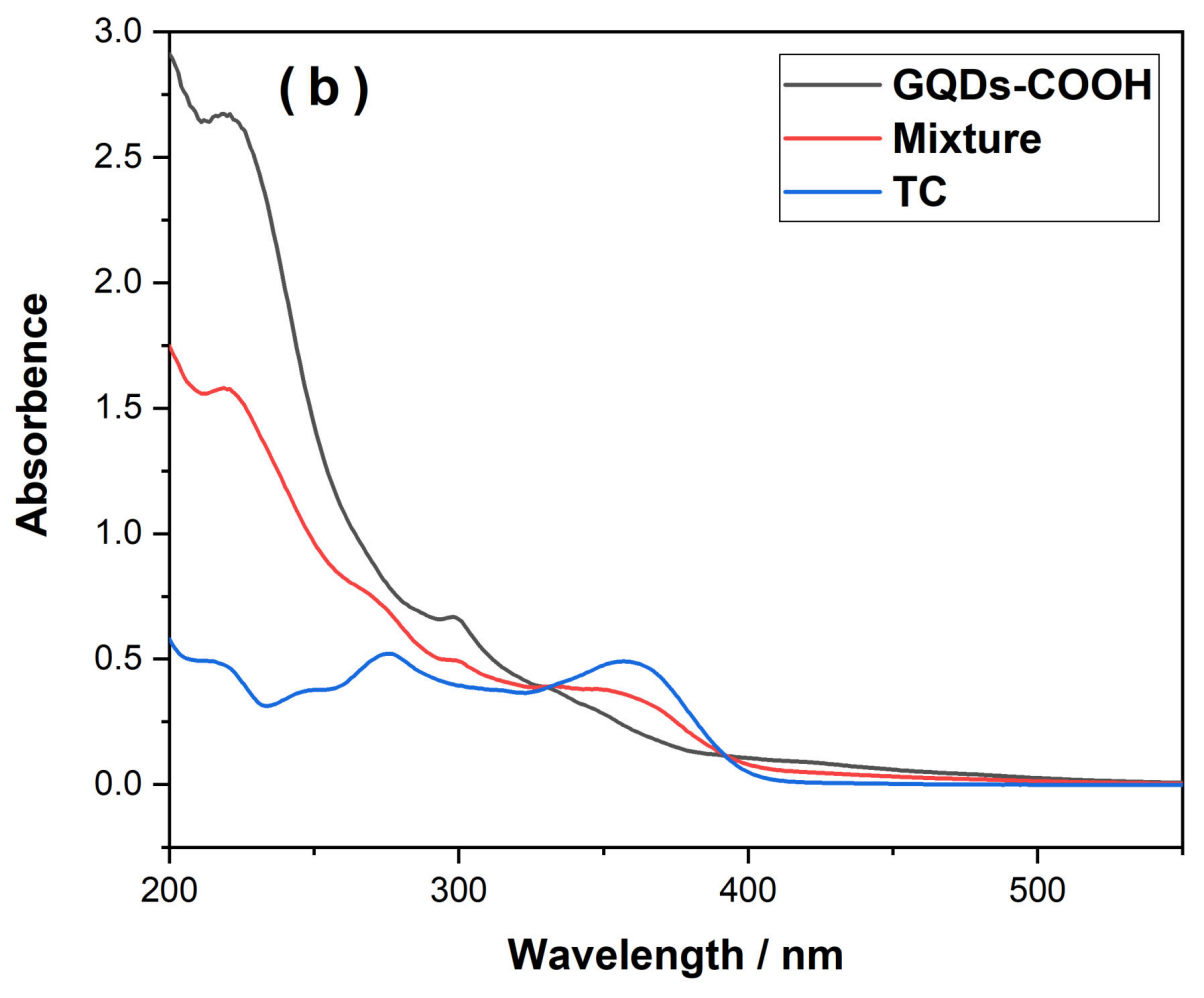
(**a**) Fluorescence emission spectra of GQDs with different functional groups with addition of TC in water. (**b**) UV spectrum of GQDs-COOH, TC and mixture.

To compare these GQDs with different functional groups, we tested the fluorescence emission spectra of GQDs with the addition of TC solutions. Quenching of GQDs-COOH was observed at the concentration at and above 10^4^ µg·L^−1^, as shown in Figure 3a. In comparison, the fluorescence of GQDs-NH_2_ could not be quenched in the presence of TC. Quenching requires molecular contact and energy transfer between the fluorophore and quencher. Here GQDs-COOH was negatively charged while GQDs-NH_2_ was positively charged. Since ammonium groups of TC were positively charged, electrostatic interaction occurred between GQDs-COOH and the template TC, which resulted in the apparent nonradiative annihilation, as reported by Chao [31]. This was further proved by the zeta potential of GQDs-COOH, which was found to be −27.6 mV in water. Therefore, MIPs based on GQDs-COOH were applied for further experiments.

To study the quenching mechanism, the absorption spectra of GQDs-COOH in the presence and absence of TC were measured as illustrated in Figure 3b. It was observed that the absorbance of GQDs changed with the addition of TC, indicating the interaction between GQDs and TC induced static quenching, as static quenching affects the absorption spectrum of the quenching molecule.

### 3.3. TEM of GQDs-MIP and GQDs

**Figure 4:**
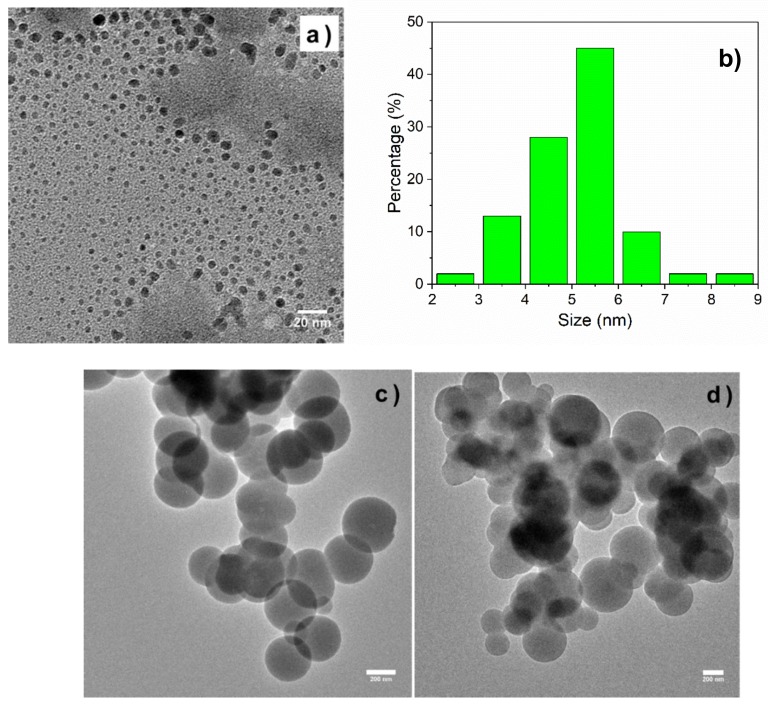
TEM images of GQDs (**a**), GQDs-MIPs (**c**), and GQDs-NIPs (**d**). The hydrodynamic size distribution of GQDs (**b**).

MIP and reference NIP were successfully synthesized by sol-gel process. The morphology and size distribution of GQDs, GQDs-MIPs and GQDs-NIPs were investigated by TEM, as illustrated in Figure 4. The GQDs had a good size distribution around 5 nm while both the GQDs-MIPs and GQDs-NIPs exhibited a size distribution with diameters in the range of 200–300 nm. After the coating of MIPs on GQDs, it is clear that the particle’s diameters increased significantly. 

### 3.4. Determination of TC Using GQDs-MIPs 

**Figure 5:**
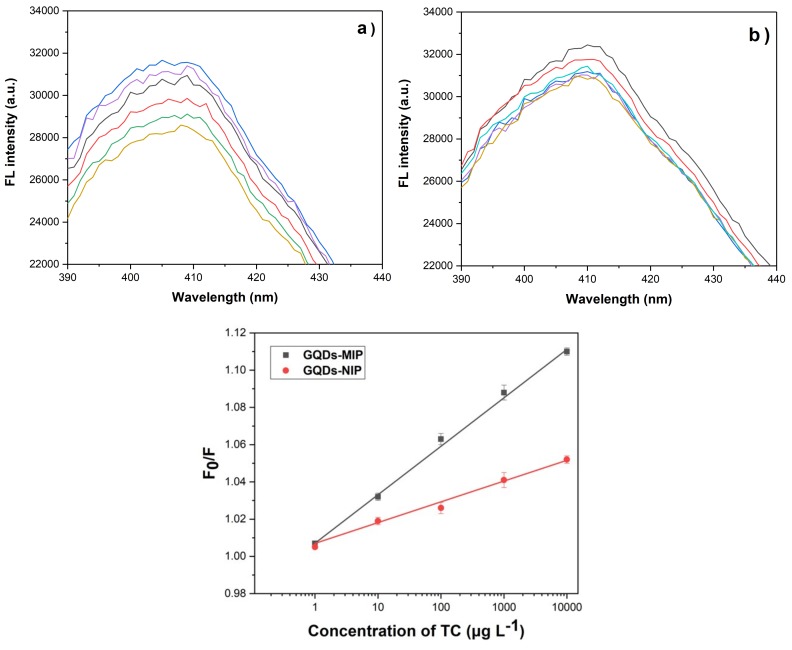
Fluorescence emission spectra of GQDs-MIPs (**a**) and GQDs-NIPs (**b**) with the addition of various amounts of TC in water. F_0_ and F represent the fluorescent intensities before and after the addition of TC solution, respectively.

To study the recognition ability of GQDs-MIPs, the fluorescence intensity of GQDs-MIPs and GQDs-NIPs were investigated at different concentrations of TC, which are shown in Figure 5a,b. Although both GQDs-MIPs and NIPs showed fluorescence quenching, the intensity of GQDs-MIPs decreased more significantly in response to the increase of TC concentration. It is clear that specific cavities of MIPs resulted in the higher quenching efficiency of GQDs-MIPs. The results showed that the GQDs-MIPs exhibited a linear quenching for the TC detection in the range of 1.0–10^4^ µg·L^−1^, and the limit of detection for TC was determined to be 1 µg·L^−1^ under optimal conditions (Figure 5c). Normally, a certain incubation time is needed for sufficient interactions between analytes and GQDs-MIPs. It is worth noting that here all tests were measured right after TC was mixed with GQDs-MIPs without incubation, suggesting that the response time of the developed sensor was very short and the quenching occurred rapidly. This is very helpful for fast detection of TC in real samples.

### 3.5. Selectivity of GQDs-MIPs

**Figure 6:**
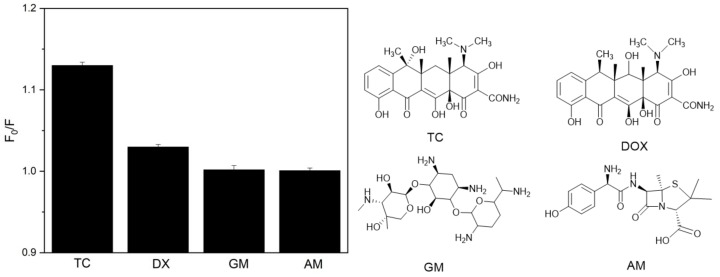
Selective adsorption of TC, DOX, GM and AM by GQDs-MIPs in water. The concentration of all compounds was 10^4^ µg L^-1^.

The selectivity of the GQDs-MIPs was further investigated by fluorescence quenching response to analog analytes. One interference compound was doxycycline (DOX), a synthetic antibiotic derived from TC. The chemical differences between them are in the substitutes of some carbon atoms, as shown in Figure 6. Because of the effectiveness of molecularly imprinting, we found that the template TC bound more strongly to the imprinted sites than DOX, and more significant changes in the fluorescence intensity of GQDs-MIPs were observed. Due to a big structure difference, the quenching of GQDs-MIPs by Gentamicin (GM) and amoxicillin (AM) were quite small. The above results revealed that GQDs-MIPs exhibited a selective recognition and excellent fluorescence quenching response toward TC. As the molecular size and shape are very similar between TC and DOX, the position of the functional groups is the major dominating factor for a specific fluorescence quenching response.

### 3.6. Real Sample Analysis

To further demonstrate the selectivity and sensitivity of GQDs-MIPs, detection and quantification of TC in real milk samples were conducted. According to the results shown in Table 1, the spiked recoveries of TC were ranging from 85.3% to 103.3%, which suggested that the combination of GQDs and MIPs was an effective method for analyzing antibiotic residues in complex food samples.

## 4. Conclusions

Nowadays, rapid and efficient detection methods for the trace amounts of antibiotics in food samples are in urgent need for food security screening. In this work, GQDs were fabricated via a simple and green synthesis approach, and the effect of functional groups on fluorescence quenching was investigated. A GQDs-MIPs-based fluorescence nanosensor was then successful prepared by the simple sol-gel method. The results showed fast detection and selectivity. Compared with natural bioassay, the fluorescent sensor has higher stability and lower cost. The work suggested that GQDs-MIPs could be widely used to screen and analyze TC in complex food samples.

## Figures and Tables

**Table 1:** Recovery of TC from spiked milk samples.

Spiked Amount (µg·L^−1^)	Measured Amount (µg·L^−1^)	Recovery (%)
1	0.98 ± 0.049	98 ± 4.9
10	9.22 ± 0.57	92.2 ± 5.7
100	103.3 ± 3.7	103.3 ± 3.7
10^3^	(9.74 ± 0.72) × 10^2^	97.4 ± 7.2
10^4^	(8.53 ± 0.61) × 10^3^	85.3 ± 6.1
